# Offering HCV treatment to prisoners is an important opportunity: key principles based on policy and practice assessment in Europe

**DOI:** 10.1186/s12889-018-6357-x

**Published:** 2019-01-08

**Authors:** H. Stöver, F. Meroueh, A. Marco, K. Keppler, P. Saiz de la Hoya, R. Littlewood, N. Wright, F. Nava, F. Alam, S. Walcher, L. Somaini

**Affiliations:** 1 Institute of Addiction Research, Frankfurt University of Applied Sciences, Frankfurt, Germany; 2 Health Unit, Villeneuve les Maguelone, France; 3 Penitentiary Program, Catalan Health Institute, Barcelona, Spain; 4 Justizvollzug, Berlin, Germany; 5 Health Services of Fontcalent Penitentiary Centre, Alicante, Spain; 6 Applied strategic, London, UK; 7 Spectrum Community Health CIC, Wakefield, UK; 8 University of Modena and Reggio Emilia, University Hospital Policlinico of Modena, Modena, Italy; 9 Divisional Medical Director, Central and North West London NHS Foundation Trust, London, UK; 10 CONCEPT, Addiction Medicine, Munich, Germany; 11 Addiction Treatment Centre, Biella, Italy

**Keywords:** Hepatitis C virus, Treatment, Prisoners, Policy, Practice

## Abstract

**Background:**

Prisoners have a high prevalence of hepatitis C virus (HCV) infection but may find it difficult to access healthcare services. This may be related to risk behaviour including history of injecting drugs and marginalisation related to problem drug use/ opioid use disorder (OUD). Direct-acting antiviral products with superior efficacy and safety compared to interferon-based regimens offer HCV cure. Many citizens in Europe have been treated, although few received therapy in prisons.

**Methods:**

Analysis of prisoner HCV treatment need and policy determinants of clinical practice was completed for 5 EU countries. Evidence was collected from national statistical sources and peer-reviewed publications to describe prison populations and HCV prevalence, to map national prison/ HCV health policy or guidance. A consensus of important principles for prisoner HCV care was developed.

**Results:**

Data from published sources describing prisoner HCV prevalence is limited. Prisoner population requiring HCV treatment is not known; estimated numbers based on analysis of evidence: England and Wales, 9000, France, 8000, Spain, 6000, Italy, 6000, Germany, 6000. Treatment access: national law defines right to equivalent care in all countries implying access to HCV therapy in prison similar to community; useful prisoner HCV guidance facilitating treatment decisions present in: 4 of 5 national/ regional HCV policy documents, 4 of 5 national prison healthcare policies. Four of five had practical prison HCV clinical guidelines. Despite existence of policy, implementation of guidance, and so HCV treatment, is suboptimal in many locations.

**Conclusions:**

Prison is an important location to detect, address and treat HCV infection in people who may be underserved for healthcare and find it difficult to navigate community treatment pathways. This is often related to problems with OUD and resulting social inequity. HCV management in prisons must be improved. Policy and clinical practice guidance must be set to promote treatment, and practical steps to make treatment easy should be followed including education to promote engagement, set-up of optimal screening and work up processes with modern tools to reduce time needed/ achieve efficiency; programs to make it easier to get specialists’ input include remote working and nurse-led services.

## Background

Chronic hepatitis C virus (HCV) infection is prevalent in people with a history of injecting drugs [1–3] – many of these people have a history of opioid use disorder (OUD) [4]. Individuals with OUD and a history of injecting drugs are often marginalized [4, 5] and face social [6], economic [7] and health problems [8]; many are underserved for healthcare with reduced access to services in the community [2, 9]. Involvement with criminal behaviour and imprisonment often becomes part of the career of people with OUD [10, 11]. Prisoners may be more likely to engage in a range of risk behaviour related to transmission of HCV and other blood borne-viruses [12, 13]. Harm reduction services, such as prison-based needle and syringe programs (PNSPs) and treatment for OUD, are important in reducing HCV prison incidence [14], and improving overall health outcomes [13]. In Spain, some prisoners have access to PNSP, but coverage remains limited in other European countries [14].

In Europe, prevalence of HCV infection in prison populations is estimated at 15% [15]. HCV treatment regimens based on direct-acting antiviral (DAA) medicines have improved efficacy, safety and tolerability profiles compared to historical interferon-based regimens [16]. Twelve DAA medicines have received European regulatory approval since 2011; access to safe and effective treatment has gradually improved for different groups [17]. Despite progress many prisoners still do not receive adequate assessment and opportunity for HCV treatment [16, 18]. Historically, many prisoners may not have engaged with HCV services due to a lack of understanding of new treatment options and concerns about the limited chance of successfully accessing treatment [19]. Organisation and infrastructure can make treatment difficult with delays associated with screening, workup and referral for treatment [19]. Access to specialists able to prescribe DAA often limits treatment. Prisoners with sentences less than 6 months may not be considered despite the shorter treatment duration of 2–3 months as a result of limited continuity of care options on release [20].

To date prisoner HCV treatment is limited in many countries in Europe; access to care is highly variable and often limited in prisons in Germany, Italy, France and England. Policy and clinical practice alignment has resulted in HCV treatment of prisoners in certain regions of Spain: national prison service, 2361 [21]; Catalunya, 529 [22]; Valencia 379 [23]. In these countries there are examples of individual prisons or regions where prisoner HCV treatment has been successful; it is observed that HCV treatment is not uniformly and easily available to all prisoners. Limited access to HCV care for prisoners is a missed opportunity as prison represents a point of care for citizens, in some cases, otherwise out of contact with conventional healthcare services.

Policy and effective implementation is a key determinant of the day to day practice in prisoner HCV care. Analysis and comparison of HCV treatment population sizes in prisons and policy determinants of clinical practice can inform the development of principles for improving prisoner HCV care in Europe.

## Methods

A structured approach to assess the size of prison populations requiring HCV treatment in selected EU countries (England, France, Germany, Italy, Spain) was followed. Evidence describing the size and HCV prevalence of prison populations was identified in a search of published literature from 2007 to 2017 in the PubMed database. Search terms included: “hepatitis C”, “HCV”, “prisons”, “prisoners”, “prevalence”. Search returned 357 results, 343 were excluded because they did not meet the selection criteria (204 not in relevant countries of study focus, 121 not focused on assessing HCV prevalence, 14 not in prison population, 4 with no abstract available). The 14 sources from search were combined with 14 additional sources from relevant national or international agencies (England 2, France 2, Germany 2, Italy 3, Spain 5). These additional sources were identified by the authors, local experts with extensive relevant experience in prisoner healthcare in each country. Risk of bias was assessed by a previously existing tool for prevalence studies review [24]; results summarized in Appendix. To manage potential bias studies conducted in a small number of prisoners or in limited number of prisons at regional not national level, or limited by lack of randomization in sampling were compared to other evidence and a consensus from authors expert in local prisoner healthcare, agreed. The size of the prison population potentially requiring HCV care was synthesised based on population size and HCV prevalence evidence.

Mapping of prisoner HCV policy and practice guidance was completed using a common approach for selected countries. Evidence was identified in a standard review of sources. Sources included the publications of relevant government departments (including health and justice) describing evidence of policy or guidance for clinical practice for prisoners or HCV-related healthcare. Responsibility and governance for prisoner HCV care was identified and recorded from these sources. Policy assessments were based on the review of sources which described: (1) relevant law defining the principle of equivalence of access to healthcare for prisoners, (2) implications of existing national HCV care plans including guidance specifically in relation to any recommendation on practice in prisons or importance of prisoner care, (3) components of national prison strategy related to prisoner HCV diagnosis and management and also (4) clinical practice guidelines specific to HCV care in prison. Data from all sources were extracted and assessed by two reviewers familiar with comparative analysis and the therapy area. A consensus on important principles for policy and practice development was developed based on the results of the analysis.

## Results

### The size and nature of the prisoner HCV population

HCV prevalence among prisoners varies across countries assessed, ranging from 5 to 43% (Table 1).

**Table 1:** A structured review of published literature estimating HCV prevalence in prisoners in 5 European countries

Country	HCV prevalence		Year of study	Study type	Sample Size, N	N, Prisons	Note	Author, Year (ref)
	Ab (%)	RNA (%)						
Spain	12.8	9.9	2017	Cross-sectional	821	1	Cantabria – El Dueso prison	Crespo 2017 [49]
	Nr	9.1	2017	Regional screening	7051	10	Cataluña	Gencat 2018 [50]
	11.0	8.8	2016	Cross-sectional	1264	10	Cataluña	Marco 2017 [51]
	18.7	11.0	2016	Aggregation of regional statistics	48,830	nr	National data used	Ministry of Interior 2017 [52]
	17.6	9.9	2016	Cross-sectional	204	3	Andalucía	Tellez 2017 [53]
	22.7	20.1	2008	Cross-sectional	378	18	Spain	Saiz de la Hoya [54]
	34.2	nr	2008	Cross-sectional	730	1	Spain	Murcia 2009 [55]
England	18.0	15.0	2014	HJIPS opt out testing programme	8797	112	England	Public Health England 2017 [43]
	8.0	nr	2014	PHE Sentinel surveillance	4089	Multiple, figure not mentioned	Data not specified	Public Health England 2015 [56]
	43.0	28.0	2013–2014	Observational	102	1	Northumberland	Darke 2015 [57]
	19.0	nr	2012	Cross-sectional	4904	14	Scotland	Taylor 2012 [58]
	11.0	nr	2010–2011	Retrospective analysis	118	1	Oxfordshire	Duncan 2013 [59]
	24.0	11.0	2005–2008	Retrospective analysis from sentinel survey	9965	39	England; (*N* = 1490* Ab+ tested for RNA)	Kirwan 2011 [60]
France	15.0	nr	2017	Survey	950	1	Maison d’Arrêt de Villeneuve-les-Maguelone	Meroueh 2017 [61]
	5.0	2.0	2012–2013	Cross-sectional	342	2	Puy-de-Dome	Jacomet 2016 [62]
	4.8 (Women 11.8; Men 4.5)	2.5	2010	Cross-sectional, questionnaire-based	1876	27	Prisons stratification by region and prison type for selection	Semaille 2013 [63]
	13.0	nr	2008	Survey	nr	1	Maison d’Arrêt de Villeneuve-les-Maguelone	Meroueh 2008 [64]
	5.0	nr	2004–2010	Cross-sectional questionnaire (multiple time points)	5957	3	Southeastern France	Roux 2014 [65]
	5.0	nr	2000–2003	Cross-sectional	597	1	Caen	Verneuil 2009 [66]
Italy	Nr	7.4	2014	Survey, questionnaire-based	15,751	57	6 regions: Toscana, Lazio, Veneto, Liguria, Umbria, ASL Salerno	Agenzia Regionale di Sanità dellaToscana [67]
	22.8	nr	2009	Cross-sectional	2241	9	Representative sample of prisons selected based on geographical location	Sagnelli 2012 [68]
	22.0	19.0	2006	Cross-sectional	695	1	Milan-Opera	Brandolini 2013 [69]
	28.0	nr	2001–2002	Cross-sectional	1091	3	Cassino, Frosinone, Latina	La Torre 2012 [70]
	9.0	nr	2006–2009	Cross-sectional	na	na	Toscana	Voller 2011 [71]
Germany	16.0	nr	2006–2007	Cross-sectional survey analysis	1582	6	Chemnitz, Hameln, Köln, Remscheid, Rheinbach, Zeithain	Eckert & Weilandt 2008 [72]
	16.0	nr	2006–2007	Cross-sectional (Serology + questionnaire)	1519	6	Closed German institutions	Radun 2007 [73]
	14.0	nr	2006	Survey, questionnaire-based	14,187	31	Based on return of completed survey from prison physicians	Schulte 2009 [74]
	9.0	7.0	2002	Cross-sectional	1125	1	German Young Offenders’ Institution	Meyer 2007 [75]

*nr* not reported

Prison populations were described including analysis of sentence length. The size of potential group of prisoners requiring treatment for HCV was estimated using sentence length to define addressable populations. A sentence length of 1 year, assuming this implies a custody period of 6 months, was considered sufficient time to allow for completion of HCV treatment in prison. Applying sentence limits to prisoner populations reduces the size of the cohort potentially able to receive HCV treatment by 50% for the selected countries. The analysis suggests a total of 35,000 (range: 25–45,000) prisoners potentially requiring HCV treatment across the 5 countries.

### Policy assessment, governance & funding: prisoner HCV care

The results of the policy assessment are summarised in Table 2. The Ministry of Health and relevant parts of the national health services are responsible for prison healthcare provision and funding in England, France and Italy at the national level. In Germany, the 16 Ministries of Justice are responsible for funding and provision of general healthcare services in prison. In Spain, in autonomous regions of Catalunya and Country Basque, funding and provision of healthcare in prisons is the responsibility of the regional health service and in the rest of the regions is the Ministry of Interior [25]. In Germany, the level of decision making, funding and provision of healthcare in prisons is devolved to the 16 regions or Länder. In all cases systems for prison healthcare provide reimbursement, in a similar way to the general population, for HCV testing and access to DAA treatment for prisoners able to navigate the journey to treatment. There is consistency of the law that defines the rights of prisoners to equivalent healthcare in all countries assessed (Spain [26], England [27], France [28], Italy [29], Germany [30]).

**Table 2:** Assessment of prisoner HCV treatment need and policy determining care in 5 European countries

	Country				
	Spain^a^	England & Wales	France	Italy	Germany
Population					
Total prisoners (‘000 s)	60 [76, 77]	86 [78]	79 [79]	56 [80]	64 [81]
Prisoners requiring HCV treatment (‘000 s)^b^	6	9	8	6	6
Prisoners, custody sufficient for treatment ‘000 s, (%)	4 (70) [82]	5 (60) [78]	4 (50) [79]	3 (50) [83]	3 (50) [81]
Policy					
Law defines equivalence of prison healthcare (Year introduced)	1996 [26]	2003 [27]	1994 [28]	1998 [29]	2009 [30]
HCV plan includes prisoners	Harm reduction; HCV Screening, Prevention & Diagnosis in prison [31]	Increase uptake & accessibility to HCV testing & treatment [32]	Harm reduction; Screening; DAA treatment; medical/ social coverage on release [33]	No HCV plan identified	Improve prevention, diagnosis and treatment of HCV [84]
Prison health plan includes HCV	Access to NEP; repeated HCV screening (inc pre-trial); HCV treatment; in situ specialist; continuity of care [40]	HCV testing on entry, during imprisonment; results provided to community-based GP, if consented [85]	Access to screening readily on entry & continuity of care [36]	Multidisciplinary team to promote awareness and provide support for HCV-infected inmates [38]	No prison health plan identified
Prisoner HCV clinical practice guidelines	All inmates offered HCV RNA test, liver biopsies without delay [41]	Access to opt-out DBS testing; in situ BBV specialist; referral & treatment via integrated care team [35]	Recommended annual HCV screening; access to DAA treatment with continuity on release/ transfer [86]	Increase HCV awareness & risk behaviour; access to screening & treatment [87]	No prisoner HCV clinical practice guidelines identified
Practice					
Access to treatment	Widespread	Increasing but limited in some areas	Increasing but limited in some areas	Increasing but limited in some areas	Limited
Governance					
Organization responsible for prison healthcare	Ministry of Interior	Ministry of Health	Ministry of Health	Ministry of Health	Ministry of Justice
Level of decision making	Regional	National	National	National	Regional
Funding	Ministry of Justice	NHS	NHS	NHS	16 Ministries of Justice

This table provides an estimate of the number of prisoners requiring treatment for HCV infection in 5 European countries and a description of the policy and governance which defines the approach to prisoner HCV care

^a^Spain has 2 Penitentiary Administrations: the Penitentiary Administration of Catalonia and the Penitentiary Administration of the rest of Spain. The data presented is the sum of those of both Administrations

^b^Prisoner HCV population was estimated based on published data sources and experience of experts; the range of data points retrieved from published sources is quoted in brackets for reference

When comparing results for the selected countries, there are differences in other relevant policy and guidance related to prisoner HCV care. A national HCV plan that specifically addressed prisoners as a key risk group is identified for Spain [31], England [32] and France [33]. Prison healthcare policy defining effective HCV care and published clinical guidelines to facilitate the day-to-day treatment of HCV in prisons were available in England [34, 35], France [36, 37], Italy [38, 39] and Spain [40, 41]. Policy and guidance likely to lead to progressive, effective clinical practice includes: explicit statement of the rights of prisoners to access to DAA therapies in the context of the development of harm reduction programs with campaigns to screen/educate on HCV risk and actions to ensure medical/ social coverage on release [33].

Relevant examples of effective policy and practice choices include the offering of voluntary HCV screening on entry and after 6 months continuously until release if the result is negative for prisoners and pre-trial detainees [40]. Opt-out HCV screening (rather than opt-in) is effective [42, 43] and use of simple tools such as dried blood spot testing kits is optimal [35, 44, 45]. Best policy and practice also includes clear referral pathways to access treatment delivered by integrated multidisciplinary care teams [35, 45–47]. Set up of services for each prison including a realistic, non-limiting, working relationship with a specialist expert in infectious diseases is advised [46, 48]; achieving continuity of care on release is also an important part of policy and guidance for services [45].

## Discussion

Advances in HCV treatment and increasing access in the community setting to HCV care have led to a step change in treatment and outcomes; many prisoners do access HCV care. Treatment of HCV for prisoners is limited in Germany, increasing but limited in some areas in England, France, and Italy. In Spain there is wider access to care with still further progress needed.

Analysis of prison populations in 5 selected countries in Europe identifies 35,000 (25–45,000) prisoners potentially requiring HCV treatment. In each country estimated prisoner HCV populations identified are England and Wales, 9000 (range: 5–13,000), France, 8000 (4–12,000), Spain, 6000 (6–8000), Italy, 6000 (3–6000), Germany, 6000 (6–8000). Evidence describing prisoner HCV population is often limited and varying data is reported. Number of patients requiring treatment in this work is estimated based on an interpretation of the available studies and experience of experts actively engaged in the treatment system for each respective country. The number is an estimate based on best available sources and it is reasonable to assume from assessing evidence from published sources identified here. The range of population size is stated based on available sources; in each case the most likely value for a country was determined by final expert decision. Experts have extensive experience in prison healthcare and the evidence of appropriate population size.

There is important opportunity to improve outcomes for this group for whom access to care can be limited. Policy development and implementation of optimal clinical practice with improved engagement is key for success (e.g. providing education alongside streamlined, efficient screening programs using modern tools to reduce inconvenience and provide essential diagnostic information without excess delay). Sentence or custody length may present a barrier for prisoner assessment and inclusion in HCV care programs, which can be addressed. Policy and clinical guidelines should ensure that there is equity of selection for prisoners and that sentence length is not used inappropriately to limit treatment. In addition, optimizing treatment process to reduce time and facilitate treatment continuity can make treatment a reality for another 15,000 prisoners with short periods of custody. The right to prescribe modern DAA therapy should be as wide as possible to address access to specialists. Learning from the approach to HIV medicines prescribing is important – non-physician prescribing should be considered where locally possible including models of oversight to enable other HCP to commence therapy.

Successful treatment of HCV in the prisons is based on clear policy defining equity of access, linked to an integrated treatment approach, which directly addresses potential barriers to success. In locations where policy and treatment approach are aligned and optimal, many prisoners with HCV have been treated and prison acts as an important contact point for HCV care engagement.

The following principles should be considered as a key part of the approach to providing treatment and improving prisoner HCV care:

1. Develop and update policy through national and/or relevant regional (1) HCV care guidelines and (2) prison healthcare guidance to include screening, testing, and treatment of HCV for prisoners
2. Adopt innovative, local clinical practice guidance including choices to improve engagement and screening, and to make the prisoner HCV treatment as easy and effective as possible
3. Implement standards and metrics for measuring and reporting activity and outcomes of prisoner HCV screening and treatment
4. Plan for integrated care models between the community and prison with healthcare record sharing to make treatment continuity the norm during prisoner release
5. Endorse a holistic approach to prisoner health including equitable access to integrated treatment programs for OUD.

Providing a treatment service in prisons consistent with these principles defines a gold standard. In this analysis no prisoner with HCV across any one country achieves full access to HCV care in prisons. Based on observations, it is noted that care is very limited in many areas in Germany, sometimes limited but increasing with areas of success in some areas in Italy, France and England and more commonly available in regions of Spain. All prisoner health services should aim to reach the gold standard of HCV care.

This work is based on an assessment of current approaches to prisoner HCV care from 5 countries in Europe. It is likely that these observations are applicable elsewhere: further work to assess the situation in other locations is recommended. Additional input from prison health, OUD and HCV specialists from other countries can add further to the understanding; many of the challenges and successes identified here may apply generally. Consensus on the size of relevant populations is reached based on the available evidence and expert familiarity with the treatment area. It is likely that the consensus represents a strong estimate of population size but this approach is limited - further measurement of prevalence of HCV in prisons should be undertaken in all settings to confirm the validity or improve this approach.

## Conclusion

Periods of custody in prison are an opportunity for people underserved for healthcare to engage with services and to provide diagnosis, workup and treatment for HCV. Many do not receive HCV assessments and treatment in prison despite their needs. This qualitative analysis shows there is an opportunity to clarify relevant policy, develop specific guidance and support day-to-day clinical practice in prisons to improve care and use this opportunity. It is important to broaden access, make screening and treatment easier to improve outcomes for prisoner HCV care in many countries in Europe. This is a key part of the challenge to treat all persons whether in prison or the community with HCV. Further work to update policy and improve access and clinical practice should follow to realise this significant opportunity.

## Appendix

**Table 3:** Analysis of risk of bias

Author	External validity				Internal validity						
	Target population is a close representation of the national population	Sampling frame a true or close representation	Some form of random selection	Minimal likelihood of nonresponse bias	Data collected directly from the subjects	Have an acceptable case definition	Study instrument that measured the parameter valid reliable	Same mode of data collection for all subjects	Length of the shortest prevalence period appropriate	Numerator and denominator for the parameter appropriate	Have a summary item on the overall risk of study bias
Crespo 2017 [49]	–	+	–	+	+	+	?	?	?	+	–
Gencat 2018 [50]	–	+	–	?	?	?	?	?	+	?	–
Marco 2017 [51]	–	+	+	?	–	+	?	?	–	+	–
Ministry of Interior 2017 [52]	+	+	?	?	+	+	+	+	+	+	–
Tellez 2017 [53]	–	+	+	+	+	+	+	+	?	+	–
Saiz de la Hoya [54]	+	+	–	+	+	+	?	+	?	+	–
Murcia 2009 [55]	–	+	–	+	+	+	+	+	?	+	–
Public Health England 2017 [43]	+	+	–	–	+	+	+	+	+	+	–
Public Health England 2015 [56]	+	+	?	?	+	+	+	+	+	?	–
Darke 2015 [57]	+	–	?	?	+	?	+	+	+	+	–
Duncan 2013 [59]	–	+	–	?	+	+	+	+	+	+	?
Kirwan 2011 [60]	+	+	?	+	+	+	+	+	+	+	+
Meroueh 2017 [61]	–	+	?	?	–	+	+	+	+	+	–
Jacomet 2016 [62]	–	–	–	–	+	+	+	+	+	+	+
Semaille 2013 [63]	+	+	+	+	+	+	+	+	+	+	+
Meroueh 2008 [64]	–	–	?	?	+	+	–	+	?	?	–
Roux 2014 [65]	–	–	–	+	+	+	+	+	+	+	+
Verneuil 2009 [66]	–	+	+	–	+	+	+	+	–	+	+
Agenzia Regionale di Sanità dellaToscana [67]	+	+	+	+	+	+	+	?	+	+	+
Sagnelli 2012 [68]	+	+	–	+	+	+	+	+	–	+	+
Brandolini 2013 [69]	–	+	–	+	+	+	+	+	+	+	+
La Torre 2012 [70]	–	+	–	?	+	+	+	+	+	+	+
Voller 2011 [71]	?	?	+	?	?	?	?	?	+	?	?
Eckert & Weilandt 2008 [72]	+	+	?	+	+	?	–	+	+	+	+
Radun 2007 [73]	+	+	–	+	+	?	+	+	+	+	?
Schulte 2009 [74]	+	+	+	–	–	+	+	+	+	+	+
Meyer 2007 [75]	–	+	–	+	+	+	+	+	+	+	–

+ (Yes) represents low risk of bias, − (No) represents high risk of bias,*?* (Unclear) represents uncertain risk of bias due to insufficient information

## Abbreviations

DAA: Direct acting antivirals
HCP: Healthcare professional
HCV: Hepatitis C virus
OUD: Opioid use disorder
PNSP: prison-based needle and syringe programs
